# Assessment of the healthcare burden of dengue disease in England: a retrospective analysis (2010–2023)

**DOI:** 10.1093/jtm/taag059

**Published:** 2026-07-02

**Authors:** George Kassianos, Larry Goodyer, Eduardo Eugênio Bittencourt de Gomensoro, Umesh Doobaree, Tracy Leong, Jennifer A Davidson, Bojana Milovanović

**Affiliations:** British Global and Travel Health Association, London NW1 2FB, UK; Leicester School of Pharmacy, De Montfort University, The Gateway, Leicester LE1 9BH, UK; Takeda Pharmaceuticals International AG, Zürich, Switzerland; Takeda UK Ltd, London, UK; Takeda UK Ltd, London, UK; Thermo Fisher Scientific, London, UK; Takeda Pharmaceuticals International AG, Zürich, Switzerland

**Keywords:** dengue, cohort study, healthcare burden

## Abstract

**Background:**

Dengue virus is transmitted by *Aedes* mosquitos, which are now prevalent in destinations commonly visited by UK travellers. Increased international travel and factors including climate change contribute to the global increase in dengue cases. Here, we evaluate the healthcare burden of dengue in England.

**Methods:**

This retrospective, descriptive, cohort study (2010–2023) utilized data collected from National Health Service healthcare records in the Clinical Practice Research Datalink Aurum, linked with Hospital Episode Statistics, and Office for National Statistics mortality data. The primary objective was to determine the occurrence of hospitalized dengue. Other objectives were evaluation of healthcare resource utilization and costs, characteristics of hospitalized dengue cases, and complications due to dengue. Exploratory cohorts for suspected dengue in primary care were also reported.

**Results:**

Data from Clinical Practice Research Datalink-linked records for 596 patients comprising the hospitalized dengue cohort were evaluated; the incidence rate of hospitalized dengue progressively increased from 2010 until the highest in 2019 (5.8 per million person-years); rates increased in 2022 after COVID-19 lockdowns. Approximately one-third of hospitalized dengue cases were aged 18–29 years, most were White or Asian, and the most common complication was gastrointestinal conditions (gastroenteritis/colitis; 34.7 cases per 1000 person-years). Healthcare costs in the year following hospitalized dengue cases were mostly driven by inpatient admission costs; ~20% of cases had a subsequent hospital admission (~50% were dengue-related). A total of 2286 suspected cases of dengue were reported in primary care, with 1066 unconfirmed and 1220 confirmed cases.

**Conclusions:**

Increasing incidence of dengue cases in England has followed global trends. The associated healthcare burden of imported dengue suggests a need for targeted public health strategies, particularly among younger travellers. Improvements in general practitioners’ reporting of suspected dengue cases and associated details of symptoms and travel history are warranted.

## Introduction

Dengue virus is a member of the *Flaviviridae* family transmitted by *Aedes aegypti* and *Aedes albopictus* mosquitos, which are prevalent in popular tourist destinations including Southeast Asia and Latin America.^1^^,^^2^ Ongoing geographical expansion of the *Aed*es mosquito has increased dengue risk in nonendemic areas including France, Italy, Spain, Portugal, and Germany.^3–5^ In recent years, mosquito surveillance activities have identified *A. aegypti* and *A. albopictus* eggs at separate locations in the UK.^6^

Dengue generally presents as a mild febrile illness; however, it can result in severe illness. Complications such as bleeding, encephalitis, Guillain-Barré syndrome, hepatitis, mononeuropathies, plasma leakage, and seizure, which can have fatal outcomes, have been reported.^7–9^ Furthermore, long-term sequelae include fatigue, chronic pain, musculoskeletal pain, liver function abnormalities, and cardiovascular complications.^10^^,^^11^ Individuals with underlying chronic diseases, such as diabetes, obesity, and hypertension, are at a greater risk of severe dengue disease requiring hospitalization.^1^^,^^12^ Limited studies have investigated the occurrence of hospitalization due to dengue with varying hospitalization rates reported by geography. Recent German analyses reported hospitalization rates among individuals with dengue to be between 37 and 53%.^13^^,^^14^ A review of GeoSentinel records from 2007 to 2022 of dengue among travellers residing in nonendemic countries found that over one-quarter of individuals with dengue were hospitalized.^15^ While in the USA, hospitalization rates were reported at 42% in returning travellers, compared with 15% in locally acquired cases.^16^

Data from the UK are limited, with statistics generated by the UK Health Security Agency (UKHSA) only reporting overall case notification numbers, which was 904 dengue cases in 2024.^17^^,^^18^ Most individuals were documented to have travelled to Southern and Southeast Asia.^18^ Presentation of dengue is rare and, like other travel-related infections, geographically dispersed. As a result,^17^^,^^18^ healthcare professionals in the UK lack awareness about dengue infection and outcomes.^19^ Greater understanding of the clinical and economic burden of dengue, as well as other mosquito-borne infections, on the healthcare system in England could raise awareness among healthcare professionals and travellers, leading to better implementation of preventive measures including vaccination. Here, we provide an estimate of the recent occurrence of hospitalized dengue cases in England and associated healthcare resource utilization (HCRU) and costs. The occurrence of suspected and confirmed dengue infection in the primary care service and care pathways for patients with suspected dengue infections in primary care is also reported.

## Methods

### Study design

This retrospective, descriptive, observational study utilized data collected from primary and secondary National Health Service (NHS) healthcare records using the Clinical Practice Research Datalink (CPRD) Aurum database, Hospital Episode Statistics (HES) from admitted patient care, outpatient, and accident and emergency datasets, and Office for National Statistics (ONS) mortality data.

### Data sources

The CPRD Aurum database of anonymized primary care electronic health records is widely used for public health research.^20^ Among the English population, ~16 million people were registered (24.2% of population), with a median follow-up time of 9.6 years (interquartile range, 3.3–22.3) at practices contributing to the dataset.^21^ Record linkage conducted by NHS England allowed the inclusion of other data sources including HES, and mortality data from the ONS.^22^ Socioeconomic data were linked through the Index of Multiple Deprivation, which provides an aggregate measure across seven domains, including income, employment, and health grouped into a single deprivation quintile for research inclusion.^23^ The HES dataset collates reimbursement records for all inpatient, outpatient, and emergency admissions to NHS hospitals in England.^24^^,^^25^ Records include patient demographics (including ethnicity), medical diagnoses at discharge [coded using International Classification of Diseases, 10th Revision (ICD-10)], and procedures (coded using Operational Procedure Classification System, 4th Edition [OPCS-4]).^25^^,^^26^ ONS mortality records are derived from death registrations, with the cause(s) of death coded using ICD-10.^27^

### Study cohorts

#### Hospitalized dengue cohort (primary analysis)

The primary analysis used a cohort of patients hospitalized with dengue. Patients of any age hospitalized for dengue were identified using ICD-10 codes from CPRD-linked HES records from 1 January 2010 to 31 March 2023 (see study flow in Supplementary Fig. S1). Follow-up data were collected through 31 March 2023 to allow for lag in linked datasets. Analysis of this data was limited to cases on or before 31 December 2022 to allow for a 3-month follow-up period after hospitalization. The index date was defined as the date of first overnight hospital admission with a dengue ICD-10 code (Supplementary Table S1). Follow-up periods varied for different outcomes; for death, the follow-up period was 90 days after index, for HCRU and costs it was 365 days after index, and for clinical outcomes it was defined as the earliest date of death, transfer out of practice, or last data collection from practice. Data were not restricted to only those collected within General Practitioner (GP) registration times.

#### Hospitalized dengue (supplemental analysis)

As analysis of the occurrence of hospitalized dengue in the primary analysis cohort was limited to cases between 1 January 2010 and 31 December 2022 (to allow for a 3-month lag in linked data following hospitalization) for the CPRD-linked HES data. A supplemental analysis, using unlinked HES data, was undertaken to provide data for an extended period between 2019 and 2024.

#### Primary care cohorts (exploratory analyses)

Exploratory evaluations were undertaken for two primary care cohorts (patients with suspected and subsequently confirmed dengue, or suspected but unconfirmed dengue). The cohorts for the exploratory analyses are given in the Supplementary Material (full description, Supplementary Table S1, Supplementary Fig. S2). Briefly, any patients with a recorded test for dengue in the CPRD-linked population were included.

#### Inclusion criteria

A list of ICD-10 and Medcode code types for inclusion criteria is provided in Supplementary Table S1. Predefined Medcodes were used to identify tests performed for dengue. The initial general inclusion criteria are that records are deemed to be of research quality by CPRD and eligible for linkage to HES Admitted Patient Care (APC) and OP and ONS datasets. The main study population cohort (hospitalized dengue) met the following criteria: earliest overnight admission to hospital coded with ICD-10 A90, A91, or A97 (and all subcodes) in HES APC dataset in the eligibility window. The exploratory study population cohorts (dengue tested or diagnosed in primary care) comprised two cohorts. The suspected dengue and not confirmed in primary care cohort met the following criteria: CPRD Aurum recorded (Systemized Nomenclature of Medicine [SNOMED] coded) test for dengue with negative or missing result during active registration at primary care practice and no subsequent dengue diagnosis Medcode. The suspected dengue and confirmed in primary care cohort met the following criteria: CPRD Aurum recorded test for dengue with positive result during active registration at primary care practice OR dengue diagnosis Medcode during active registration at primary care practice.

### Objectives and endpoints

The first primary objective was to estimate the occurrence of hospitalized dengue in England from 1 January 2010 to 31 December 2022 from CPRD-linked HES records. Annual incidence rates were calculated as the number of patients with first hospitalization for dengue in the eligibility period in the respective calendar year (numerator), divided by the total person-time contributed in each calendar year by CPRD Aurum patients who were eligible for linkage to HES (denominator). Annual incidence rates of hospitalized dengue were stratified by age group and ethnicity.

The second primary objective was to estimate primary and secondary HCRU and their associated costs. The number and proportion of all-cause (i.e. any reason or condition) and dengue-related [i.e. ICD-10 code specific to dengue or a complication of interest (see Supplementary Table S2 for list)] hospital admissions were calculated to estimate HCRU- and cost-related outcomes for 365 days following (including index) dengue hospitalization. All-cause hospital admissions were defined as the number of admissions among patients with at least one admission post index admission, with the proportion calculated using the total number of patients with hospitalized dengue as the denominator. Dengue-related hospital admissions were defined similarly (as the number of patients with at least one additional dengue admission post index admission) using the same denominator. HCRU and cost metrics associated with hospitalized dengue were captured as those for inpatient, primary care, and outpatient and emergency care (Supplementary Table S3). Costs were inflated to 2020/2021 pounds sterling using the NHS Cost Inflation Index, allowing costs to be summed.

Secondary objectives included evaluation of baseline demographics and clinical characteristics of the hospitalized dengue population. Outcomes evaluated were age, age group, sex, ethnicity, deprivation quintile (as a measure of socioeconomic status), geographic region, comorbidities, pregnancy status, hospitalized dengue prior to the study period, dengue symptoms in the 2 months preindex, and the number of GP practices at which patients who had hospitalized dengue within the last 2 years of the study period were registered. Additionally, complications that occurred among hospitalized dengue cases were described. Acute complications were those occurring 2 weeks preindex and chronic complications occurring 3 months post index (Supplementary Table S2).

For the supplementary analysis of the unlinked HES dataset to generate annual incidence rates for 2019 to 2024, ONS mid-year population estimates for England were used as the denominator.

Exploratory objectives utilized CPRD-linked HES records and included incidence rates of suspected (and confirmed or unconfirmed) dengue in primary care (calculated as the annual rate of suspected dengue with a negative test result, or suspected dengue with a subsequent positive test result or dengue diagnosis overall, stratified by age group and geographic region) and care pathway of patients with dengue (Supplementary Table S4).

### Approval

This study was approved by the Research Data Governance Process for CPRD research who have ethical approval from a National Research Ethics Service Committee for all purely observational research using anonymized CPRD data and standard linked datasets (study reference ID 24_004335).

## Results

Between 1 January 2010 and 31 December 2023, 49 585 087 patients were identified in the CPRD, and of those, 34 219 002 were eligible for HES and ONS linkage. Among these, 596 patients had an overnight hospital admission coded to relevant ICD-10 codes in the HES admitted patient care dataset between January 2010 and March 2023.

### Hospitalized dengue incidence rates

Among the 596 patients who comprised the hospitalized dengue cohort, the number of dengue cases and the incidence rate of hospitalized dengue increased from 2010 onward and was highest in 2019 (5.8 per million person-years) and lowest in 2020 (0.9 per million person-years) during the COVID-19 pandemic (Fig. 1A).

**Figure 1:**
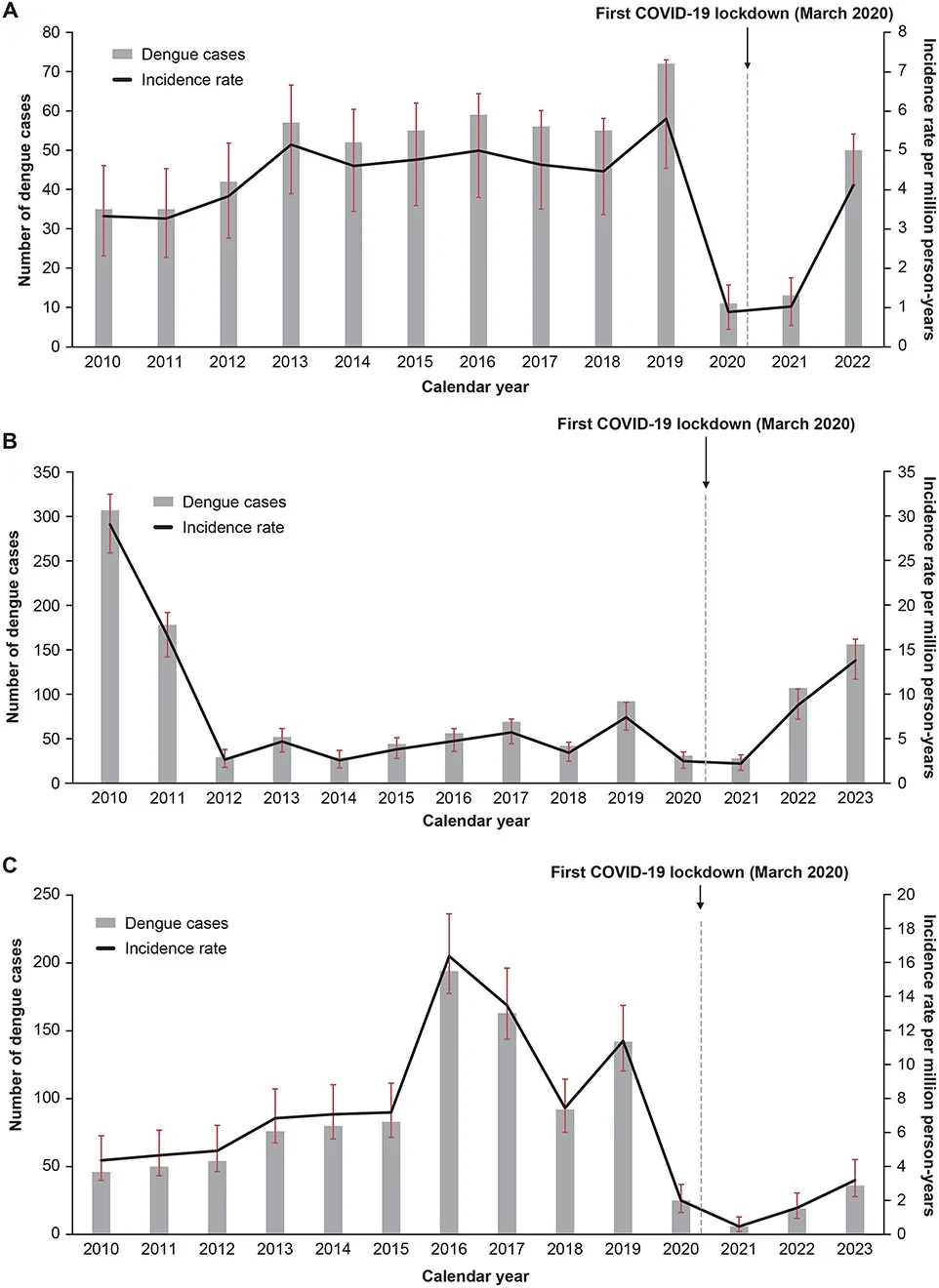
Incidence rates and number of dengue cases for the (A) hospitalized dengue cohort, (B) suspected and confirmed dengue in the primary care cohort, and (C) suspected and unconfirmed dengue in the primary care cohort.

A supplementary analysis of data from HES records (not linked with the CPRD) for patients hospitalized with dengue between 2019 and 2024 showed that incidence rates of hospitalized dengue cases were lowest in 2020 at 0.5 per million person-years but rose to around 5 per million person-years in 2024 (Supplementary Fig. S3).

Both datasets (CPRD-linked and HES records only) showed rising dengue cases from 2022 onward after the COVID-19 lockdowns in 2020 and 2021.

### Primary care dengue incidence rates

Of the 2286 suspected dengue cases reported in primary care settings between 2010 and 2023, 1220 were confirmed dengue cases and 1066 were unconfirmed (negative or unknown test result). Incidence rates of dengue during 2010 to 2023 are shown for the suspected and confirmed dengue cases in Fig. 1B and for the unconfirmed cases in Fig. 1C.

### Baseline demographics and clinical characteristics

Baseline demographics and clinical characteristics of the hospitalized dengue cohort (CPRD-linked data) and primary care suspected dengue cohorts are shown in Table 1. Some minor patient overlap between the hospitalized cohort and the exploratory cohorts was expected but not quantified. Baseline characteristics were generally similar between the hospitalized dengue and primary care cohorts, although the suspected and unconfirmed dengue cohort was older, with a higher proportion of patients that were least deprived and White than the other two cohorts. In the hospitalized dengue cohort, peak hospitalizations occurred among patients aged 18–29 years (Supplementary Fig. S4).

**Table 1:** Baseline demographics and clinical characteristics of the hospitalized dengue (primary) and the suspected dengue and confirmed/unconfirmed in primary care setting (exploratory) cohorts.

*n* (%)	Primary cohort	Exploratory cohorts	
	Hospitalized dengue (*n* = 596)	Suspected dengue and confirmed in primary care (*n* = 1220)	Suspected dengue and unconfirmed in primary care (*n* = 1066)
Age, years			
Median (IQR)	33 (24–46)	37 (26–52)	42 (30–55)
<18	35 (5.9)	152 (12.5)	62 (5.8)
18–29	195 (32.7)	248 (20.3)	204 (19.1)
30–39	161 (27.0)	254 (20.8)	221 (20.7)
40–49	90 (15.1)	201 (16.5)	208 (19.5)
50–59	64 (10.7)	185 (15.2)	196 (18.4)
≥60	51 (8.6)	180 (14.8)	175 (16.4)
Sex			
Female	284 (47.7)	614 (50.3)	560 (52.5)
Male	312 (52.3)	606 (49.7)	506 (47.5)
Ethnic group			
Asian	204 (34.2)	441 (36.1)	265 (24.9)
Black	30 (5.0)	82 (6.7)	43 (4.0)
White	327 (54.9)	624 (51.1)	711 (66.7)
Mixed or other	24 (4.0)	40 (3.3)	22 (2.1)
Indices of multiple deprivation^a^			
1 (least deprived)	105 (17.6)	186 (15.2)	311 (29.2)
2	126 (21.1)	230 (18.9)	244 (22.9)
3	135 (22.7)	381 (31.2)	225 (21.1)
4	143 (24.0)	300 (24.6)	211 (19.8)
5 (most deprived)	87 (14.6)	123 (10.1)	75 (7.0)

^a^Indices of multiple deprivation rank on local level geography assigned into quintiles. Indices of multiple deprivation are measures of relative deprivation based on income, employment, education, health, crime, housing, and environment to rank areas from least to most deprived.

IQR, interquartile range.

Among the hospitalized dengue and primary care cohorts, a range of baseline comorbidities were also reported (Table 2). Gastrointestinal conditions (gastroenteritis or colitis, calculus of gallbladder or bile duct) were most frequently reported among all cohorts.

**Table 2:** Baseline comorbidities of the hospitalized dengue (primary) and the suspected dengue and confirmed/unconfirmed in primary care setting (exploratory) cohorts.

Comorbidities, *n* (%)	Primary cohort ^a^	Exploratory cohorts (primary care) ^b^	
	Hospitalized dengue (*n* = 596)	Suspected dengue and confirmed (*n* = 1220)	Suspected dengue and unconfirmed (*n* = 1066)
Gastrointestinal condition^c^	121 (20.3)	295 (24.2)	292 (27.4)
Prior bleeding	32 (5.4)	55 (4.5)	73 (6.8)
Anaemia	28 (4.7)	75 (6.1)	57 (5.3)
Cardiovascular disease	26 (4.4)	57 (4.7)	51 (4.8)
Diabetes (type 1 or 2)	25 (4.2)	82 (6.7)	70 (6.6)
Neurological condition	24 (4.0)	55 (4.5)	57 (5.3)
Renal disease	15 (2.5)	70 (5.7)	46 (4.3)
Immunodeficiency	15 (2.5)	46 (3.8)	55 (5.2)
Connective tissue disorder	11 (1.8)	14 (1.1)	18 (1.7)
Prior hospitalized dengue (excluding index encounter)	9 (1.5)	71 (5.8)	16 (1.5)
Peptic ulcer disease	5 (0.8)	16 (1.3)	12 (1.1)
Blood-borne virus	5 (0.8)	14 (1.1)	13 (1.2)
Chronic pulmonary disease	NA^a^	12 (1.0)	9 (0.8)

^a^Acute respiratory distress syndrome, liver failure, chronic pulmonary disease, peripheral arterial disease, paraplegia, and dementia were explored, but results were suppressed due to low frequency.

^b^Acute respiratory distress syndrome, liver failure, paraplegia, peripheral arterial disease, and dementia were explored, but results were suppressed due to low frequency.

^c^Gastrointestinal condition defined as gastroenteritis or colitis or calculus of gallbladder or bile duct.

NA, not available.

### Dengue symptoms

The most common dengue symptoms for both the hospitalized dengue and primary care cohorts were fever and myalgia, although the proportion of patients experiencing dengue symptoms was relatively low (<12%) in all three cohorts, with no notable differences between cohorts (Supplementary Table S5).

### Hospitalized dengue cohort: complications

Among the hospitalized dengue cohort (*n* = 590), gastrointestinal conditions (gastroenteritis or colitis) were the most frequent acute complications, with 34.7 cases per 1000 person-years reported (Fig. 2). For chronic complications, myalgia (6.9 per 1000 person-years) and type 2 diabetes (4.2 per 1000 person-years) were most frequently reported.

**Figure 2:**
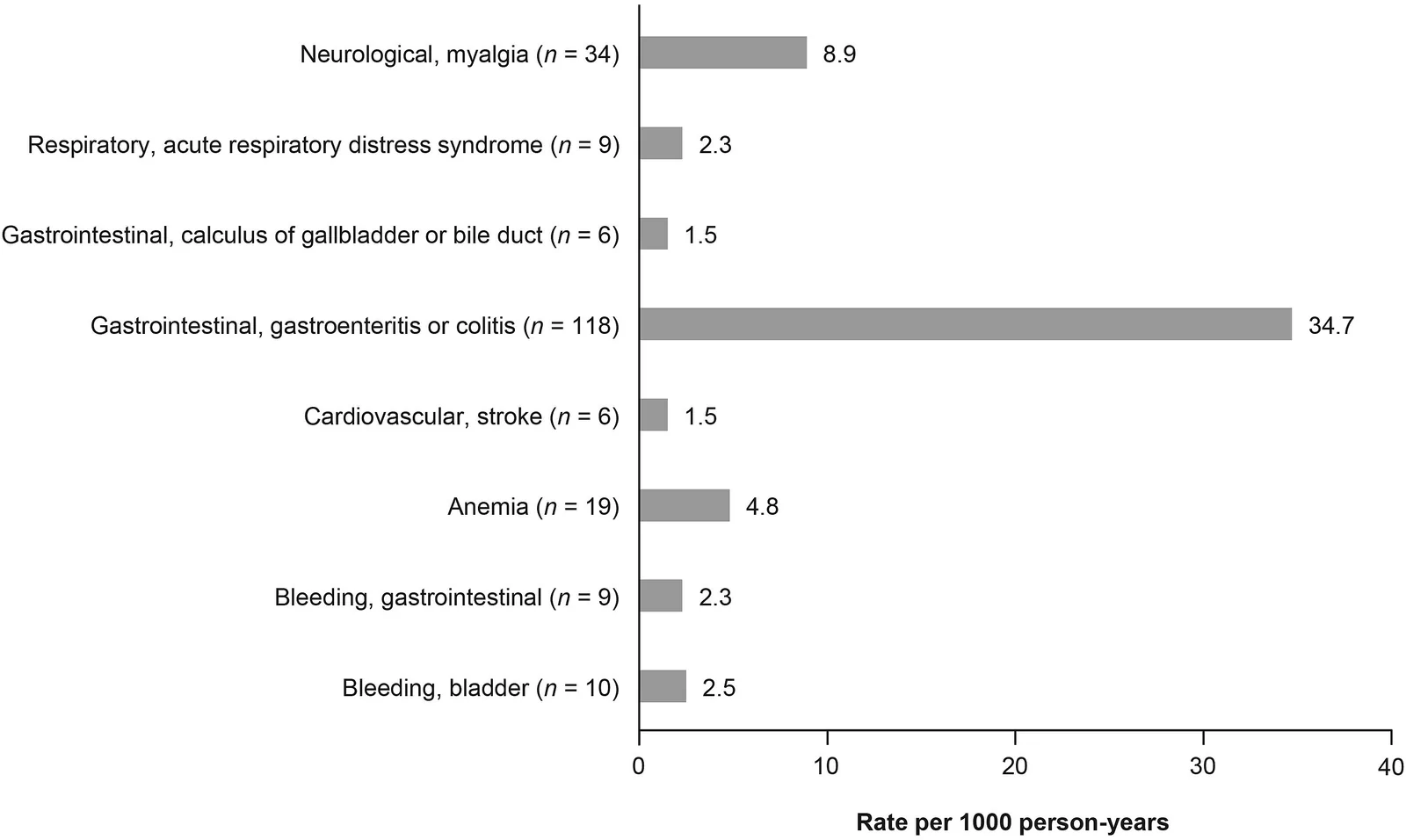
Acute complications among the hospitalized dengue cohort (*N* = 590).

### Hospitalized dengue cohort: HCRU and costs

Among the cohort who attended a primary care setting during the year following hospitalized dengue (*n* = 228), 185 (81.1%) had at least one primary care attendance in the year subsequent to hospitalization [mean six visits per year at a mean (SD) cost of £150.90 (£179.00) per patient] and 37 (16.2%) for dengue-related causes [mean (SD) 0.4 (1.5) visits and mean (SD) cost of £10.72 (£34.30) per patient] (Fig. 3A). Healthcare costs in the year following hospitalized dengue were mostly driven by inpatient admission costs (Fig. 3B). Mean (SD) dengue-related inpatient admission costs were £1899.00 (£3765.40) per index hospital event; other costs for dengue-related care were minimal. The mean (SD) length of hospital stay was 4 (7.7) days for index (dengue) admission. Mean all-cause and dengue-related primary and secondary care costs by year from 2019 to 2023 are shown in Supplementary Fig. S5.

**Figure 3:**
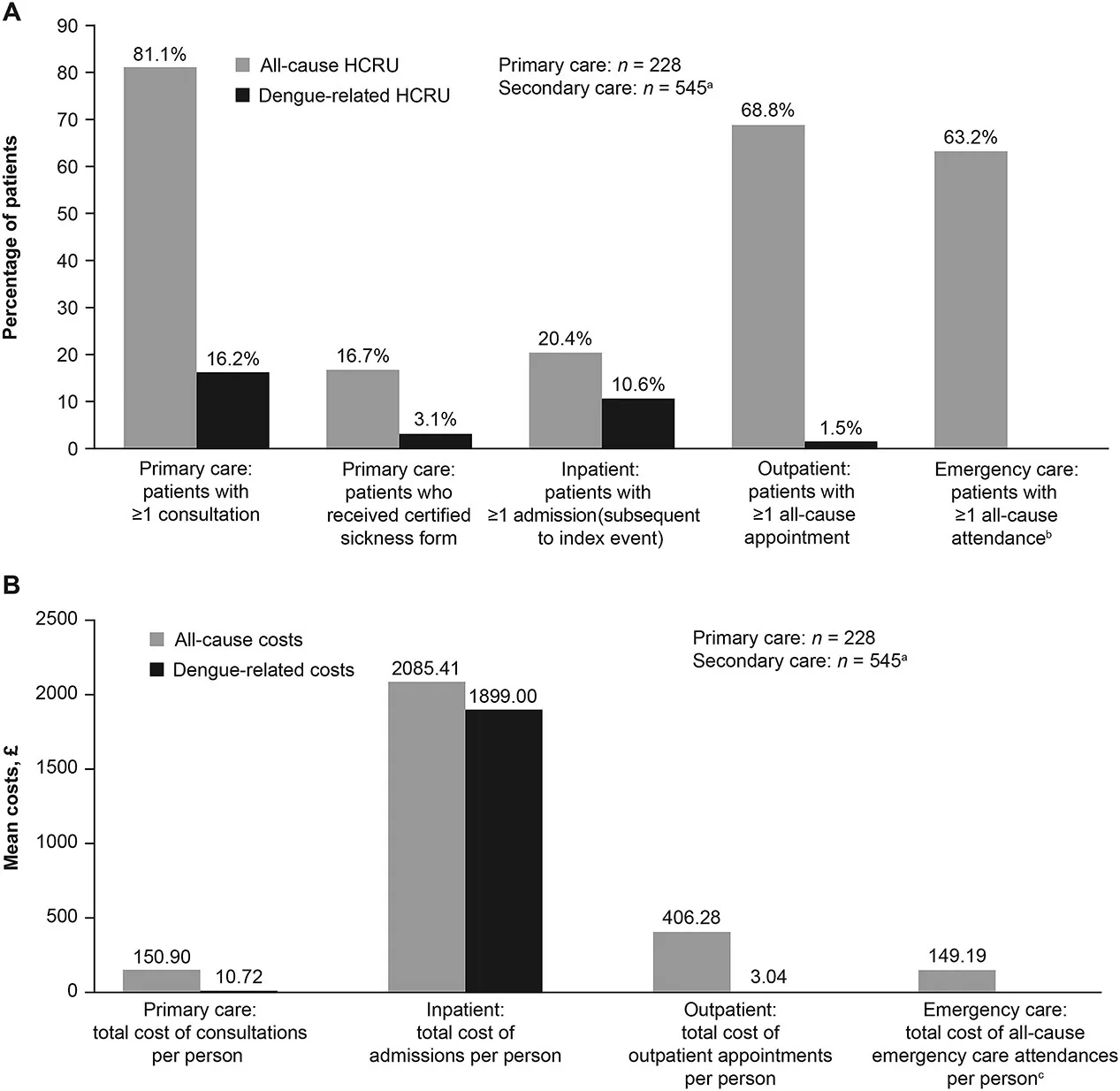
All-cause and dengue-related (A) HCRU and (B) costs following hospitalized dengue from index date until 1 year post index. ^a^Reduced data availability for emergency care, *N* = 478. ^b^Dengue-related data not available for emergency care. ^c^Cost metrics are identified in Supplementary Table S3.

#### Primary care dengue cohorts: care pathways

In a primary care setting, the mean (SD) number of dengue tests was 1 (0.38) per person in the suspected and confirmed cohort and 0 (0.31) per person in the unconfirmed cohort. Among the 1064 patients in the suspected and confirmed cohort, 9.6% (*n* = 102) presented to both primary and secondary care; of these, 64.7% (66/102) presented to secondary care first. The mean (SD) time between first primary care and subsequent secondary care presentation was 1.67 (2.88) days. Among the 1030 patients in the suspected and unconfirmed cohort, 2.5% (*n* = 26) presented to both primary and secondary care; of these, 57.7% (15/26) presented to secondary care first. The mean (SD) time between first primary care and subsequent secondary care presentation was 3.0 (5.66) days.

## Discussion

We investigated the occurrence of hospitalized dengue, and associated HCRU and costs in England for cases reported between 2010 and 2023. The annual incidence rates of hospitalized dengue varied over time from 0.5 to 3.9 per million person-years, with the lowest rate in 2020 likely due to COVID-19 pandemic–related disruption, with a rebound from 2022, aligning with the global resurgence in dengue seen post pandemic.^17^ In the UK, the number of reported cases has risen from 631 in 2023 to 904 in 2024,^17^ the highest recorded up to that point, with all cases associated with foreign travel. Despite the relatively low number of cases, the occurrence of dengue among travellers is relatively high compared with other travel-related infections, such as Chikungunya or Zika virus.^18^ Malaria cases are also rising: UKHSA reported a 26% increase in number of cases from 2022 to 2023 (*n* = 2106).^28^

Among the 596 patients identified with hospitalized dengue, approximately one-third were young adults. The most commonly reported baseline comorbidity was gastrointestinal conditions. Rates of acute and chronic dengue complications were low; similarly, the most common acute complication was gastrointestinal conditions (gastroenteritis or colitis), while myalgia was the most common chronic condition. There is limited published information regarding gastrointestinal complications associated with dengue; however, it may be that these manifestations are considered atypical or are underreported because of a lack of awareness that these symptoms are due to dengue.^29^^,^^30^ Conversely, people might present with gastrointestinal symptoms post travel and dengue could be identified following that. Myalgia is also a well-known complication among patients with dengue that can persist.

Despite the relatively low number of hospitalized dengue cases in the present study, 20% of those with hospitalized dengue had a subsequent inpatient admission. Nearly 70% had at least one outpatient appointment (all causes), and more than 80% had at least one primary care attendance (all causes) within 1 year of hospitalization. These outcomes resulted in a reasonably high (given the age profile of the population) level of HCRU and costs; these were considered to be for any causes but may be linked to clinical complications of dengue.

Among the ~2300 patients in the primary care cohorts who presented with suspected dengue, ~53% had confirmed dengue. The annual incidence rate for this cohort peaked at 29.1 per million person-years in 2010, declining during the COVID-19 pandemic. The peak in 2010 may be due to outbreaks of dengue and the cumulative increase in global cases. Furthermore, since 2010, there have been reports of autochthonous cases in Europe, including in Croatia, France, Israel, Italy, Portugal, and Spain.^2^ Among the suspected and confirmed dengue cohort in our study, a minority of patients presented to both primary and secondary care settings. For the suspected but unconfirmed cohort, the annual incidence rate peaked at 16.4 per million person-years in 2016, also declining during the COVID-19 pandemic. Among this unconfirmed cohort, dengue testing was low, which may have allowed misdiagnosis or under-recognition of the disease (HES does not include codes specific to dengue testing). Overall, although these databases provide some information about hospitalized dengue, limited information is available about symptoms and complications.

A recent study in Germany noted that dengue poses a sizable health economic burden in the year following diagnosis, as it increases HCRU and costs compared with individuals without dengue.^13^ The retrospective data analysis, using an anonymized claims database representing 11% of the German population, identified 440 dengue cases diagnosed using ICD-10 codes between 2015 and 2018.^13^ Over a longer period in Germany (2014 to 2023), 887 dengue episodes were recorded.^14^

There are several limitations to our study. Data are based on hospital records where symptom recording is low, and some patients may not have been accurately coded (i.e. they may have had dengue symptoms and been admitted to hospital but not allocated an appropriate ICD-10 code for dengue disease) or their symptoms may not have had an ICD-10 code at all. Furthermore, symptoms may not have been recorded in HES datasets. Overall, this makes it challenging to have a full picture of dengue symptomatology using these data sources.

In addition, data in the Aurum database covers ~20% of the English population and may not be representative of the whole population. Although the rates of hospitalized dengue are relatively low, the denominator is the whole English population. Ideally it would be best to use the travelling population to the dengue endemic areas, but these data are not available.

## Conclusion

Given the healthcare and economic burden of dengue among travellers returning to England from endemic areas, GPs should maintain awareness of the disease in patients presenting with febrile disease meeting these criteria and be prepared to test for dengue. Improvements in GPs’ reporting of suspected dengue cases, including details of travel history, symptoms, and severity, would provide useful information for understanding the healthcare impact of dengue. The availability of dengue vaccination can be part of a potential dengue mitigation strategy^31^; however, it does not negate the need for avoiding insect bites. Finally, given that the younger population comprised the greatest proportion of hospitalized dengue cases, public health strategies to educate younger travellers about the risks for dengue, particularly for those visiting endemic countries, are warranted.

## Supplementary Material

Resubmission_BoD_in_Engl_MS_Supplementary_material_30June2026_taag059

## Data Availability

This study was conducted using data obtained under licence from the UK Medicines and Healthcare products Regulatory Agency following study protocol approval by the Clinical Practice Research Datalink (CPRD) Research Data Governance (RDG) process. Access to CPRD and linked datasets must be requested by application via the CPRD RDG process. Therefore, the dataset cannot be made publicly available. The analysis code is available upon request.
