# Supplementary material for: Assessment of the healthcare burden of dengue disease in England: a retrospective analysis (2010–2023)
Source: J Travel Med. 2026 Jul 2;33(6):taag059. doi: 10.1093/jtm/taag059 (PMC13621469; doi:10.1093/jtm/taag059)
Supplement: Supplementary file 1 [file resubmission_bod_in_engl_ms_supplementary_material_30june2026_taag059.docx]

**Supplementary material**

**General criteria for inclusion**

Both Clinical Practice Research Datalink (CPRD)-based study populations (hospitalized dengue and dengue tested or diagnosed in primary care) were limited to patients who met the following criteria:

1. Records deemed of research quality by CPRD

2. Eligible for linkage to Hospital Episode Statistics (HES) admitted patient care (APC) and outpatient and Office for National Statistics datasets.

In addition to the general criteria, the main study population cohort (hospitalized dengue) met the following criteria:

1. Earliest overnight admission to hospital coded with International Classification of Diseases, 10th Revision, codes A90, A91, or A97 (and all subcodes) in HES APC dataset within the eligibility window.

In addition to the general criteria, the exploratory study population cohorts (dengue tested or diagnosed in primary care) comprised two cohorts that met the following criteria:

***Suspected dengue and unconfirmed in primary care***

a. CPRD Aurum recorded (Systematized Nomenclature of Medicine Clinical Terms [SNOMED] coded) test for dengue with negative or missing result during active registration at primary care practice

AND

b.	No subsequent dengue diagnosis Medcode.

***Suspected dengue and confirmed in primary care***

a.	CPRD Aurum recorded test for dengue with positive result during active registration at primary care practice

OR

b.	Dengue diagnosis Medcode during active registration at primary care practice.

Note that patients with a CPRD Aurum recorded test with a negative result and subsequent positive dengue test result within 30 days of the other were included in the suspected and confirmed cohort.

***Primary care cohorts (exploratory analyses)***
Patients of any age with a recorded dengue test that had a positive result or a confirmatory dengue diagnosis identified using SNOMED within the study period (1 January 2010 to 31 December 2023) were included in the suspected and confirmed dengue cohort, while those with a recorded dengue test who had a negative or unknown result or only suggestive dengue diagnosis were included in the suspected and unconfirmed dengue cohort (**Supplementary Table S1**). For both cohorts, the index date was defined as the date of the first test for dengue or diagnosis of dengue in primary care. For patients in the suspected and confirmed dengue cohort, the index date was set to be prior to 2022 to allow for follow-up of those included during that year (patients after 2022 were not included in the study). The study flow is shown in **Supplementary Figure S2**.

Supplementary Table S1 ICD-10 and Medcode code types for inclusion criteria

| **Criteria** | **Code type** | **Specific codes (description)** |
| --- | --- | --- |
| Hospitalized dengue | ICD-10 | A90 (Dengue fever [classical dengue])  A91 (Dengue haemorrhagic fever)  A97 (Dengue)  A97.0 (Dengue without warning signs)  A97.1 (Dengue with warning signs)  A97.2 (Severe dengue)  A97.9 (Dengue, unspecified) |
| Dengue diagnosis in primary care | Medcode | 480874010 (Dengue haemorrhagic fever)  612921000006114 (Dengue haemorrhagic fever)  2607831000000114 (Dengue without warning signs)  2607871000000111 (Dengue without warning signs)  2613981000000112 (Severe dengue)  3053081000006114 (Dengue virus)  3117941000006114 (Dengue)  3117951000006111 (Dengue fever) |
| Dengue testing in primary care | Medcode | 1786450011 (Dengue nucleic acid detection)  4326151000006114 (Dengue virus antibody)  4329571000006115 (Dengue virus antigen)  459589013 (Dengue virus IgM level)  6054351000006110 (Dengue virus IgM level)  459588017 (Dengue virus IgM level)  1786795015 (Dengue polymerase chain reaction)  354121000000112 (Dengue virus serology)  2331381000000114 (Dengue nucleic acid detection assay)  2402751000000110 (Dengue virus ribonucleic acid detection assay)  1227817014 (Screening for dengue fever) |

ICD-10, International Classification of Diseases, 10th Revision; Ig, immunoglobulin.

**Supplementary Table S2** Acute and chronic complications of interest in patients with hospitalized dengue

| **Acute (occurring within 2 weeks before index date)** | **Chronic (occurring 90 days after index or until date of death, whichever date occurred first)** |
| --- | --- |
| Bleeding | Blood-borne illnesses (hepatitis B, hepatitis C, HIV) |
| Anemia | Cardiovascular conditions (atrial fibrillation, ischemic heart disease, heart failure) |
| Cardiovascular conditions (myocardial infarction, stroke, transient ischemic attack) | Diabetes (type 1 diabetes mellitus, type 2 diabetes mellitus) |
| Gastrointestinal conditions (gastroenteritis or colitis, calculus of gallbladder or bile duct) | Liver failure (end-stage liver disease) |
| Acute liver failure | Renal conditions (chronic kidney disease) |
| Acute renal failure | Neurological conditions (encephalopathy, fatigue syndrome, neuropathy, myalgia, myositis, Guillain-Barré syndrome) |
| Neurological conditions (neuropathy, myalgia) |  |
| Acute respiratory distress syndrome |  |

**Supplementary Table S3** Healthcare resource utilization and cost metrics associated with hospitalized dengue

**Inpatient**

- Number and proportion of patients with a subsequent (beyond index admission) all-cause inpatient admission
- Number and proportion of patients with a subsequent dengue-related inpatient admission
- Number of all-cause inpatient admissions
- Number of dengue-related inpatient admissions
- LOS for index inpatient admission
- LOS for subsequent all-cause inpatient admissions
- LOS for subsequent dengue-related inpatient admissions
- Number and proportion of patients with all-cause ICU admissions
- All-cause inpatient costs (excluding ICU)
- Cost of all-cause ICU admissions
- Length of stay for all-cause ICU admissions
- Dengue-related inpatient costs among patients with hospitalized dengue

**Primary care**

- Number of all-cause primary care consultations
- Number of dengue-related primary care consultations
- Number and proportion of patients with one or more secondary care referrals
- Number and proportion of patients who had a certified sickness form from their GP
- Annualized all-cause primary care consultation costs
- Annualized dengue-related primary care consultation costs

**OP and EC**

- Number and proportion of patients with all-cause OP appointment
- Number and proportion of patients with dengue-related OP appointment
- Number of all-cause OP appointments post index admission
- Annualized all-cause OP costs
- Number of dengue-related OP appointments post index admission
- Annualized dengue-related OP costs
- Number of dengue-related EC attendances post index admission
- Annualized all-cause EC costs

EC, emergency care; GP, general practitioner; ICU, intensive care unit; LOS, length of stay; OP, outpatient.

**Supplementary Table S4** Care pathways for those with suspected and confirmed/unconfirmed dengue

- The number and proportion of individuals who presented to both settings (primary and secondary care)

- Number and proportion of individuals with first presentation in each (primary or secondary care) setting

- Time in days between presentation in primary care and secondary care for patients who presented in both settings (number of days between first primary care presentation and secondary care presentation among those who presented to primary care first)

- Number of tests for dengue per person in primary care (among those who presented to primary care)

- Number of primary care attendances between first attendance in primary care to first secondary care presentation per person (among those who presented to both primary and secondary care)

- Number of secondary care presentations per person (defined as same-day emergency care visit or hospital admission due to dengue) that occurred because of primary care referrals

**Supplementary Table S5** Dengue symptoms recorded for the hospitalized dengue (primary) and the suspected dengue and confirmed/unconfirmed in primary care setting (exploratory) cohorts

|  | **Primary cohort^a^** | **Exploratory cohorts^b^** | |
| --- | --- | --- | --- |
|  | **Hospitalized dengue  (*n* = 596)** | **Suspected dengue and confirmed in primary care  (*n* = 1220)** | **Suspected dengue and unconfirmed in primary care  (*n* = 1066)** |
| Fever | 58 (9.7) | 78 (6.4) | 127 (11.9) |
| Myalgia | 29 (4.9) | 17 (1.4) | 21 (2.0) |
| Headache | 10 (1.7) | 26 (2.1) | 24 (2.3) |
| Fatigue | 14 (2.3) | 18 (1.5) | 37 (3.5) |
| Nausea | NA | 7 (0.6) | NA |
| Vomiting | NA | 9 (0.7) | 8 (0.8) |

Data are presented as *n* (%).

^a^Bleeding, nausea, vomiting, pain at back of eyes, skin rashes, and bruising were explored, but results were suppressed due to low frequency. ^b^Bleeding, pain at back of eyes, skin rashes and bruising were explored, but results were suppressed due to low frequency.

NA, not available.

**Supplementary Figure S1** Study design schematic for the hospitalized dengue cohort. ^a^The lower interval limit for baseline/covariate assessment; −∞ denotes a lookback period containing all available retrospective data. ^b^Follow-up window for case fatality outcome (death) from index date (for hospitalized dengue) until earliest of date of death or 90 days after index. ^c^Censor for healthcare resource use (HCRU) and cost outcomes defined as the earliest of date of death, transfer out of practice, last data collection from practice, 365 days after index (see study considerations), or 31 March 2023. ^d^Censor for clinical outcomes (incidence of dengue, complications, and death and treatments), defined as the earliest date of death, transfer out of practice, last data collection from practice, or 31 March 2023. ICD-10, International Classification of Diseases, 10th Revision.

**Supplementary Figure S2** Study design schematic for the exploratory primary care cohorts. ^a^The lower interval limit for baseline/covariate assessment; −∞ denotes a lookback period containing all available retrospective data. ^b^Censor for incidence rates (clinical outcome) defined as the earliest date of death, transfer out of practice, last data collection from practice, or 31 March 2023.

**Supplementary Figure S3** Incidence rates and number of hospitalized dengue cases using national Hospital Episode Statistics data only (not linked with Clinical Practice Research Datalink) from 2019 to 2024.

**Supplementary Figure S4** Hospitalization rates by age group in the hospitalized dengue cohort.

**Supplementary Figure S5** Annual trend in mean all-cause and dengue-related primary and secondary care costs among patients with hospitalized dengue, indexed between 1 January 2010 and 31 March 2022, and with active Clinical Practice Research Datalink registration at the time of dengue. All-cause costs include primary care consultations, inpatient admissions, intensive care unit stays, outpatient appointments, and emergency care visits. Dengue-related costs include any primary care consultations, inpatient admissions, or outpatient appointments with a code for dengue or any of the complications of interest. Total cost was summed for each patient and attributed to their index year even if the costs spanned into the subsequent year. Population was limited to those in active registration at a Clinical Practice Research Datalink–contributing practice at the time of hospitalized dengue to include primary care costs. Data for 14 patients were not eligible for linkage with the Hospital Episode Statistics accident and emergency dataset, and data for this dataset are only available up to 31 March 2020; therefore, some of the costs associated with emergency care will be missing from the all-cause costs summary.
